# Insights into systematic reviews and meta-analyses: Are we judgmental enough?

**DOI:** 10.3205/000359

**Published:** 2026-06-12

**Authors:** Ulas Kumbasar, Ahmet Baris Durukan

**Affiliations:** 1 Hacettepe University, School of Medicine, Department of Thoracic Surgery, Ankara, Turkey; 2 Liv Ankara Hospital, Department of Cardiovascular Surgery, Ankara, Turkey; 3 İstanbul İstinye University, School of Medicine, Department of Cardiovascular Surgery, Ankara, Turkey

**Keywords:** systematic review, meta-analysis, scientific credibility

## Abstract

Systematic reviews (SRs) and meta-analyses are regarded as the pinnacle of evidence-based medicine, synthesizing fragmented research to guide clinical decision-making. However, the exponential increase in their publication has raised concerns regarding redundancy, methodological flaws, and misleading conclusions. Many SRs lack preregistration, employ inconsistent methodologies, and fail to account for clinical heterogeneity. Additionally, the integration of artificial intelligence (AI) in research poses new challenges, including data fabrication, plagiarism, and AI-induced biases. While AI offers efficiency in processing vast amounts of data, its unregulated use may compromise research integrity. Furthermore, current review processes often overlook critical clinical insights, leading to misinterpretations. To improve SR quality, a rigorous, stepwise approach is necessary, including preregistration, comprehensive literature searches, unbiased data abstraction, and meticulous assessment of confounding factors. AI-generated content must be scrutinized through human oversight to ensure accuracy and reliability. Ultimately, the overreliance on statistical aggregation without critical clinical evaluation may lead to flawed conclusions. Clinicians must actively engage in assessing SRs beyond methodological frameworks, integrating human judgment to enhance the credibility and applicability of findings. Addressing these challenges is crucial for maintaining the integrity of evidence-based medicine.

## Introduction

Systematic reviews (SR) and meta-analyses function as key tools for clinicians in their evidence-based decision-making process. Their position has been considered at the top of the evidence-based medicine study hierarchy. They serve as a way of identifying key knowledge gaps, piecing together these fragmented and selectively reported published information for further investigation. SRs aim to assess existing knowledge on a particular subject, whereas a meta-analysis is a statistical method used to combine results from the relevant studies and resynthesize them within a larger sample size [1], [2]. Summarizing data from individual trials increases the statistical power and the precision of the effect estimates [3]. Therefore, clinical decisions could be based on the totality of the best evidence instead of the results of individual studies. 

## History of systematic reviews

The evolution of systematic reviews has significantly impacted the lives of preterm newborns suffering from respiratory insufficiency. Collaboration developed in response to Archie Cochrane’s request to the medical community to compile “a critical summary, periodically adapted, of all… pertinent randomized controlled trials.” He has initially assessed the impact of steroids on newborns and determined that steroids helped facilitate pulmonary maturation in preterm pregnancies. This has ultimately transformed the future [4]. With courage from his early work, science has shifted into another era to find out if randomized controlled studies and large series really meant scientific value. 

## Current status

The credibility of a SR relies heavily on the quality and heterogeneity of the underlying studies, as well as a rigorous assessment of bias and confounding factors guided by a transparent protocol. Unfortunately, many existing SRs and meta-analyses are undermined by redundancy, a focus on trivial research questions, or misleading claims, which ultimately limits their scientific value [5]. 

## Perspective

Our perspectives regarding this drive include the following. First, the rate of publication of SRs and meta-analyses has dramatically increased in the last decade (2,728% and 2,635%, respectively; from 1991 to 2014) [1]. This massive increase may reflect the performance of replicating previous studies. Siontis et al. demonstrated in their study that 65% of the overlapping meta-analyses on the same topic did not include any additional outcomes [6]. Second, these replicating/overlapping studies may end up with different conclusions and can be confusing. One can reach variable final conclusions by using different selection criteria of relevant studies, choosing different outcomes of interest, comparing different populations, and using different statistical methods. Even the interpretation of similar results may differ among the researchers/clinicians based on their scientific backgrounds, the ability of critical thinking, and conflicts of interest. A thorough understanding and judgmental analysis of the data is the key factor in the correct interpretation of studies. Third, systematic and transparent conduct and reporting of SRs are still uncommon. Quantitative analysis reveals that solely around 15% to 30% of SRs are prerecorded in registries such as PROSPERO. Although there has been an increase in registrations in recent years, approximately two-thirds of published SRs still bypass this critical step, significantly increasing the risk of outcome switching and redundancy [7], [8]. Lastly, most current SRs involve non-randomized studies, which make the comparison of cohorts unreliable in most instances despite efforts at conducting propensity matching and other methods of adjusting for confounders. Propensity score-based analyses account only for known and observed patient characteristics, and thus, the confounders that are not accounted for remain as a limiting factor [9]. Meta-analyses do not necessarily address this as well. Overall, the quality of evidence and the validity of findings belonging to many SRs and meta-analyses are low. 

## Use of artificial intelligence (AI) technologies

The ongoing progress of AI-driven language technology enhances the possibilities for automating the SR process through alternative approaches. Utilizing these AI-driven solutions provides an opportunity to improve the SR process, conserving time and resources while mitigating risks associated with human responses. By evaluating these alternatives, we may optimize workflows and improve the overall efficiency of conducting SR [10]. Nevertheless, this application has certain disadvantages. The use of AI in scientific research has introduced new forms of academic misconduct, mostly data fabrication and plagiarism using AI algorithms [11]. The accessibility of free web-based technology has led several individuals lacking scientific expertise to fabricate data and utilize AI for its analyses. AI technologies themselves do misconduct by copy-pasting, therefore making “enhanced” plagiarism and even fabricating references, so-called “AI hallucination”. The provided references include authors from the relevant field; the topic is significant and supposedly published in subject-related journals. Nonetheless, the reference in question is nonexistent [12]. These techniques compromise the integrity of the research in question and can readily mislead conclusions. Unfortunately, investigating such misconduct remains a significant challenge for academic institutions, largely due to the technical limitations of current detection tools. Recent studies indicate that available AI-detection software often yields low accuracy rates and high false-positive rates, making it difficult to definitively distinguish between human-written and AI-generated text [13]. As AI technology evolves at a pace that outpaces the development of reliable detection methods, institutions are left without robust tools to enforce ethical standards. Consequently, the emphasis on ethical norms and researcher qualifications risks being overshadowed as the barriers to generating sophisticated, albeit potentially fabricated, content are lowered by these rapidly developing technologies Emphasizing ethical norms and enhancing qualifications of researchers, which are main duties of communities, are already forgotten since “brilliant-minded new generation science people” have already overcome barriers with light-speed developing AI technologies. Such issues are also observed in SR and meta-analysis conduct since AI technologies are capable of writing papers even in seconds. Therefore, it is crucial to acknowledge that although AI offers invaluable assistance, researchers must rigorously assess the model’s outputs, carefully check information, and verify the accuracy and reliability of the generated content. Moreover, rapid implementation of complete criteria for AI research integrity, including precise protocols for AI utilization in data analysis and publication, is essential to ensure transparency and accountability. 

## Pros and cons of AI

Artificial intelligence (AI) offers several important advantages in the conduct of systematic reviews and meta-analyses, particularly in improving efficiency and scalability. AI-driven tools can rapidly screen large volumes of literature, assist in identifying relevant studies across multiple databases, and reduce the time required for title and abstract screening. Natural language processing algorithms can support automated data extraction from published texts and tables, while machine learning models can aid in detecting patterns, inconsistencies, or potential biases within datasets. These capabilities may enhance reproducibility and reduce human error, particularly in repetitive and labor-intensive steps of the review process. Furthermore, AI can facilitate living systematic reviews by continuously monitoring and incorporating newly published evidence, thereby keeping analyses up to date in rapidly evolving fields.

However, these advantages must be balanced against important limitations and risks. AI systems remain dependent on the quality and structure of input data and may propagate existing biases or introduce errors such as hallucinated references, incorrect data extraction, or misclassification of studies. Overreliance on AI outputs may also contribute to “automation bias,” where researchers accept algorithmic results without sufficient critical appraisal. Therefore, the integration of AI into systematic reviews should be viewed as an assistive, rather than autonomous, process. Human oversight remains essential to validate findings, ensure clinical relevance, and interpret results within the appropriate methodological and clinical context. A hybrid model combining AI efficiency with expert clinical judgment is likely to provide the most robust and reliable approach to future evidence synthesis.

## Review process

The reviewers’ aspect is an issue as outlined. Reviewing a systematic review or meta-analysis constitutes a specialized area of expertise. Misapprehensions and insufficient technical expertise may result in the early acceptance of manuscripts lacking evident scientific value. A comprehensive understanding for evaluation is needed [14].

## Suggestions

What can be done? Adopting a step-by-step approach to conducting a SR may help to improve the quality of these studies. These include (i) designing a detailed review protocol with explicit inclusion and exclusion criteria by adhering to the PRISMA statements; (ii) preregistration of full study protocol including the statistical analysis plan; (iii) performing a comprehensive systematic literature search of multiple databases as well as the unpublished data to minimize the publication bias, (iv) accurate data abstraction by at least two independent investigators; (v) careful anticipation of clinical, statistical, and methodological heterogeneity; (vi) detailed assessment of the risk of biases and confounding factors in individual studies; (vii) adequate use of statistical methods; and (viii) assessment of the overall quality of evidence (e.g. GRADE) in each study [5], [15]. Moreover, these studies should be designed and conducted by non-conflicted investigators, and the implementation of the results should be based on transparency and critical thinking. Likewise, AI-generated content must undergo assessment through human supervision by ensuring alignment with set standards, finding and correcting any potential errors or biases, and delivering a thorough and precise interpretation of the content [16]. Table 1 (Tab. 1) summarizes the clinician’s checklist for appraising SRs.

**Figure 1:**
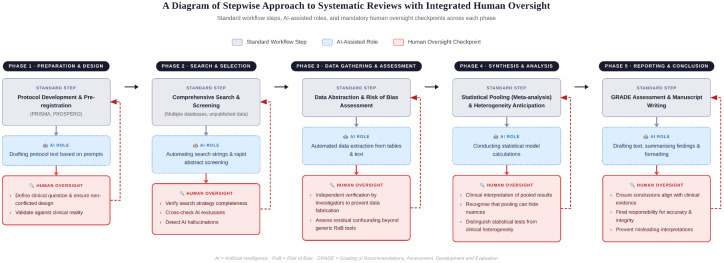
Conceptual flow diagram: stepwise approach to improving SR quality with integrated human oversight in the AI era

However, the above-mentioned methodological steps do not sufficiently capture the issue. As clinicians, we must go further. Our emphasis on the literature search or the statistical tests makes us think that this can overcome the underlying bias due to clinical issues that are not well spelled out in the papers. In that regard, the general “risk of bias” assessments do not help much either. These tools are more designed to just eliminate some studies rather than to highlight what residual confounding exists and how it might impact our interpretation. The methodologists are doing the best they can in an abstract and general manner. What they are missing is the ability to assess actual issues because they do not have any clinical insight. And the clinicians think that because the methodologists provide the criteria we just need to follow, and do not need to think critically ourselves. Furthermore, meta-analysis inherently lumps all the information together and hides the underlying nuances, and statistical tests for heterogeneity are fundamentally different than clinical heterogeneity. A diagram of stepwise approach to SRs with integrated human oversight has been proposed in Figure 1 (Fig. 1). 

## Conclusion

While systematic reviews and meta-analyses remain the cornerstone of evidence-based medicine, their current value is frequently undermined by redundancy, methodological inconsistencies, and the unregulated use of artificial intelligence. To restore the credibility of these studies, the scientific community must enforce stricter adherence to transparent protocols, including comprehensive preregistration and independent data verification. Furthermore, while AI offers efficiency, it must remain under strict human oversight to prevent the propagation of fabricated or biased data. Ultimately, researchers must recognize that statistical aggregation can obscure underlying nuances; therefore, a rigorous assessment of clinical heterogeneity must accompany statistical testing. By prioritizing expert clinical judgment alongside methodological rigor, we can ensure these studies remain reliable tools for decision-making. 

## Notes

### Authors’ ORCIDs

- Ulas Kumbasar: 0000-0003-0616-1326
- Ahmet Baris Durukan: 0000-0003-0566-0350

### Author’s contributions

- The conception of the work; or the acquisition, analysis, or interpretation of data for the work: UK, ABD
- Drafting the work, reviewing it critically for important intellectual content: UK, ABD
- Final approval of the version to be published: UK, ABD
- Agreement to be accountable for all aspects of the work in ensuring that questions related to the accuracy or integrity of any part of the work are appropriately investigated and resolved: UK, ABD

### Data availability

The data underlying this article will be shared on reasonable request to the corresponding author.

### Competing interests

The authors declare that they have no competing interests.

## Figures and Tables

**Table 1:**
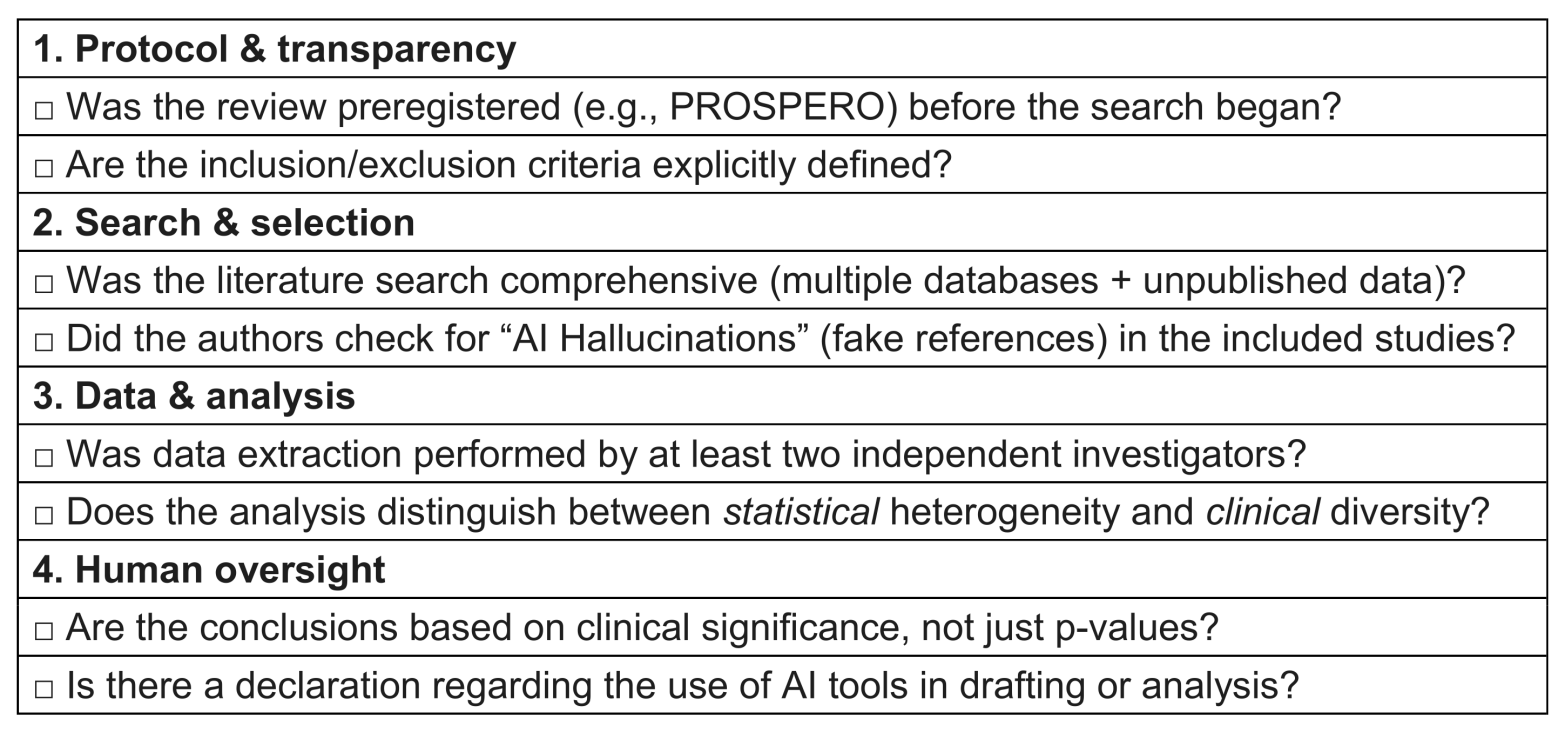
Clinician’s checklist for appraising systematic reviews
